# Capacitive resistive electric transfer modifies gait pattern in horses exercised on a treadmill

**DOI:** 10.1186/s12917-020-2233-x

**Published:** 2020-01-09

**Authors:** Mireya Becero, Aritz Saitua, David Argüelles, Antonia Lucía Sánchez de Medina, Cristina Castejón-Riber, Cristina Riber, Ana Muñoz

**Affiliations:** 1 Equine Sport Medicine Center CEMEDE, School of Veterinary Medicine, University of Córdoba, Campus Universitario de Rabanales, 14004 Cordoba, España; 2 Veterinary Teaching Hospital, School of Veterinary Medicine, Autonomous University of Barcelona, Bellaterra, 08193 Barcelona, Spain; 3 Department of Animal Medicine and Surgery, School of Veterinary Medicine, University of Córdoba, Campus Universitario de Rabanales, 14004 Cordoba, España; 4 Veterinary Teaching Hospital, School of Veterinary Medicine, University of Córdoba, Campus Universitario de Rabanales, 14004 Cordoba, España; 5 Department of Corporal Expression, University of Córdoba, 4004 Córdoba, Spain

**Keywords:** Accelerometry. Capacitive resistive electric transfer. Exercise. Horse. Locomotion. Performance

## Abstract

**Background:**

Capacitive resistive electric transfer (CRET), a radiofrequency at 448 kHz, resulted in increased superficial and deep temperature and hemoglobin saturation, faster elimination of metabolic and inflammatory products and enhanced sport performance in humans. This research aims to investigate whether the application of CRET affects the locomotor pattern in horses and to assess whether an accumulative effect appears when two CRET sessions are applied two consecutive days.

**Methods:**

Nine horses were subjected to two CRET sessions applied in both right and left sides of neck, shoulder, back and croup. The horses were exercised on a treadmill, at walk and at trot, before CRET application and at 2, 6 and 12 h after. A second CRET session was applied next day, and the animals were evaluated again at the same times (i.e. at 26, 30 and 36 h after the first session). Between 5 and 7 days later, the same horses were subjected to a sham procedure and they were evaluated in the same times as in the CRET experiment. During treadmill exercise, locomotor parameters were measured with a triaxial accelerometer fixed in the pectoral region and in the sacrum midline.

**Results:**

The sham procedure did not affect any of the accelerometric variables studied. CRET applications resulted in greater total powers, which resulted in absolute increased dorsoventral, mediolateral and longitudinal powers. However, a reduction in dorsoventral power expressed as a percentage of total power was found. Stride regularity increased. The greater total power resulted in longer stride length and because the velocity was kept fixed on the treadmill, stride frequency decreased. An accumulative effect of CRET application was only found in stride length and frequency.

**Conclusions:**

It appears that CRET is a useful technique to enhance power and to elongate the stride at defined walk and trot velocities. The effect of these changes on performance should be studied for horses competing in different sport disciplines.

## Introduction

Gait profile is a key to performance in the equine athlete. Locomotor characteristics associated with performance have been measured by accelerometry in horses competing in technical disciplines, such as dressage and showjumping, but also in competitions highly dependent on running economy, as galloping and trotting racing and endurance events [1–3]. Accelerometric analysis is a kinetic method which measures instantaneous changes of velocity, which appears as a consequence of applying a force on a solid. The use of the accelerometers allows the calculation of the acceleration on the surface on which the device is fixed. This acceleration vector is proportional to the force applied to the body in which the accelerometer has been fixed. Consequently, its measurements provide a convenient and practical, easy and low-cost way to study the kinetics of a body in motion [4].

Good galloping racehorses exhibit greater propulsive power (PP) and lower dorsoventral displacement (DVD) of the center of gravity in order to reduce the energy cost of locomotion [2, 4]. In addition, they are characterized by an optimal stride length (SL), because maximum gallop velocity (V) is primarily determined by SL. Further, the most successful racing horses are those that are able to increase their stride frequency (SF) to the highest values at the end of the race, after achieving the longest SL [2]. High PP activity and large DVD values are desirable gait characteristics in dressage horses, and positive significant correlations with better scores in performance tests have been described [3]. Fatigued endurance horses commonly experienced a reduction in V and SL [5]. As a consequence of these relationships, different strategies have been implemented in the horse in order to improve these accelerometric characteristics.

In human beings, physical therapies based on electromagnetic stimulation, specifically, a system of capacitive resistive electric transfer (CRET) has been demonstrated to affect spatiotemporal biomechanical parameters after exercise [6]. CRET delivers radiofrequency energy that passes between active and inactive electrodes, resulting in increased deep (muscle) temperature and blood flow and flexibility and improved hemoglobin saturation [7, 8]. Duñabeitia et al. [6] considered that these physiological changes would promote a more efficient running pattern, as shown by the lower SF and longer SL reached in their study after application of a CRET session in recreational runners. These authors speculated that the observed improvements in biomechanics would likely lead to improvements in performance during competitions in these runners, even though this affirmation was not proved in their research.

To the authors’ best knowledge, the effects of this type of radiofrequency on the locomotor pattern of the horse have not been evaluated yet. Based on the results obtained in previous studies in humans, the main objective of the current study was to investigate whether the application of CRET might affect the accelerometric parameters measured at walk and at trot in sound horses exercised on a treadmill. A second objective was to evaluate whether an accumulative effect appears when two CRET sessions were applied on two consecutive days. The proposed hypotheses were: 1) that the application of CRET in horses would change total power (TP) and would also lead to a redistribution of TP in the three body axes (dorsoventral, longitudinal and mediolateral); 2) probably because of these changes, stride spatiotemporal variables would also be modified and 3) an accumulative effect would appear when the sessions of CRET are applied on two consecutive days.

## Material and methods

### Horses

A total of 9 healthy adult horses, with a mean age of 11.2 ± 3.4 years (range 8–15), 2 Angloarabians and 7 Spanish horses, of both sexes (4 mares and 5 geldings), with a mean body weight of 430 ± 89 kg (range 390–495 kg) and with different level fitness, were enrolled in the study. The two Anglo-Arabian horses were in active training and regularly participated in endurance competitions of 120 km with excellent results. Of the 7 Spanish horses, 6 were used for Exercise Physiology and Equine Sports Medicine classes at the Equine Sports Medicine Center of the Veterinary School. They were exercised between 3 and 5 days per week. Exercise consisted in 1 h of walking in a walker and exercises on a treadmill (10 min walk, 10–15 min trot, 3–5 min canter and 1–2 min gallop). The other Spanish horse competed in prix St George level dressage.

The horses were subjected to clinical and lameness exams and complete hematological and biochemical tests were carried out, including fibrinogen determination. Lameness exam was performed by two experienced equine clinicians (D.A. and C.R.) and consisted in evaluation in motion (straight line, circling, inclines, hand and soft surfaces…), hoof testers and joint flexion tests. Diagnostic nerve and joint blocks were not considered necessary because only horses with a score of 0 over 5, according to the American Association of Equine Practitioners AAEP were selected for the research. Further, only horses without laboratory alterations were included.

The horses were adapted to treadmill exercise, because treadmill exercise was included in their training program during at least the last 9 months before starting the research. They were kept in medium-size paddocks and during the 2 days before and the 2 days after the study, the horses were not trained.

### Device for capacitive resistive electric transfer CRET

Indiba® Animal Health Vet905 (Indiba® S.A., Barcelona, Spain) was used for CRET application. This device operates at a frequency of 448 kHz. A rigid circular metallic electrode was used as the active electrode and a large flexible rectangular metallic plate (size: 200 × 260 mm) was used as the inactive electrode. The device delivers radiofrequency energy in two modes at the active electrode: capacitive (CAP) and resistive (RES). The CAP electrode has a polyamide coating that acts a dielectric medium, insulating its metallic body from the skin surface, generating superficial heat, near the skin. The RES electrode is uncoated and therefore, radiofrequency energy travels directly through the body into the inactive electrode, generating heat in the deeper parts of the body. The inactive electrode was placed on the sternum during CRET applications.

### CRET sessions

A manufactured supplied conductive gel was applied as a coupling medium between the electrode and the skin surface. Each session had a total duration of approximately 50 min. The protocol of the session combined the use of both types of electrodes, CAP and RES following the manufacturer’s indications. CRET was applied in both the right and left side of the animals, starting in one of the sides randomly. Bilateral epaxial musculature of the neck (M. trapezius, M. rhomboideus, M. splenius, *M. serratus* ventralisthoracics and M. brachiocephalicus), shoulder (M. supraspinatus, M. infraspinatus, M. triceps brachii), back (*M. longissimus* dorsi) and croup (M. gluteus superficialis, M. gluteus medius, M. biceps femoris, M. semitendinous, M. semimembranous) were treated in a craniocaudal direction. Each session consisted of 2 min of CAP therapy and 4 min of RES therapy. Potency was fixed at 40% for CAP therapy and at 30% for RES therapy. Due to the greater development of the musculature of the croup, the application time of the RES therapy was 5 min.

### Treadmill exercise, TE

Horses were exercised on a high-speed treadmill (Mustang 2000, Kagra®), at a constant V for each horse. Each horse was subjected to the V used habitually for training (walk: 1.5 ± 0.1 m/s; trot: 3.4 ± 0.2 m/s). Exercises were made with the treadmill without inclination, for at least 5 min each gait, in order to ensure that the horses had time to relax and to adopt a regular locomotor pattern, as recommended [9]. The accelerometric parameters were measured between the 5th and the 7th min of exercise at each gait.

The TE were performed in the following times: baseline (BL), before application of CRET; at 2, 6 and 12 h after the first session of CRET; and at 2, 6, and 12 h after the second session of CRET (i.e., 26, 30 and 36 h after the first session). In the same times, data of ambient temperature and relative humidity were obtained with a portable weather station (RTGR328N, Oregon Scientific TM wireless®).

### Accelerometric data acquisition

Accelerometric data were obtained with a portable gait analyzer (Equimetrix, Centaure-Metrix®), consisting of three orthogonal accelerometers that measure acceleration along the three body axes, a data logger and a scientific software program (Equimetrix-Centaure 3D®) for processing acceleration signals. The recorder collected data continuously at a sampling rate of 100 Hz. The accelerometer was placed in the caudal part of the sternum, between the right and left *pectoralis ascendens* muscles at the level of the girth, as previously recommended [1–4]. In this location (PECT), the device is near the body center of gravity, with a good stability against the body of the horse and provides more information about the acceleration parameters in the forelimb. Also, the accelerometric device was attached to the skin over the midline of the sacrum region (SML), using an adhesive tape in order to better analyze changes in the hindlimbs. The same researcher (M. B.) positioned the transducer in all occasions. More information about this device has been previously reported [1–4].

### Accelerometric parameters

Three groups of accelerometric parameters were studied. Stride energetic parameters include dorsoventral power DVP (W/kg), propulsion power PP (W/kg) and mediolateral power MLP (W/kg). DVP is the integral of the power spectrum obtained by Fast Fourier Transformation (FFT) from the dorsoventral acceleration signal and measures limb suspension and loading activity. PP measures the craniocaudal or longitudinal activity and it is the integral of the power spectrum obtained by FFT from the longitudinal acceleration signal. PP measures the amount of acceleration and deceleration along the longitudinal axis. MLP is the side-to-side activity, calculated as the integral of the power spectrum obtained by FFT from the lateral acceleration signal. MLP therefore, measures the amount of acceleration and deceleration along the lateral axis. The sum of the three powers (DVP, PP and MLP) reflects body kinetics [10] and it represents the total power TP (W/kg). DVP, PP and MLP were also expressed as percentages of TP in order to assess whether a redistribution of TP appears. Additionally, dorsoventral displacement (DVD) of the center of gravity was also calculated.

Stride coordination parameters included regularity, REG (dimensionless) and symmetry SYM (dimensionless). REG measures the acceleration pattern similarity of successive strides in a period of time and SYM measures the similarities between left and right acceleration patterns. Stability (STAB) of the gait was calculated as the sum of SYM and REG and it was used as an indicator of gait quality [11].

Stride spatiotemporal parameters included V (m/s), SF (cycles/s or Hz) and SL (m). V was fixed in the treadmill and SF and SL values were calculated by the accelerometer.

Each accelerometric parameter was calculated in each time of recording (7 times), for the two gaits (walk and trot), and with the accelerometer device in the two positions, PECT and SML. In each of these recordings, 6 measurements were calculated for each horse and the data are presented as the means of these 6 measurements.

### Sham procedure

Between 5 and 7 days after the CRET experiment, the same 9 horses were subjected to a sham procedure, consisting in two sessions with the same CRET device without operating, on the same anatomical areas and during the same period of time (50 min). Accelerometric recordings were carried out at the same times as the CRET group (BL, at 2, 6, 12, 26, 30 and 36 h after the first session).

### Statistics

Analysis of data was made with a commercial statistic software (Statistica v. 12.0 for windows). A Shapiro-Wilk test was used to assess for normality. As W value was lower than 0.05, the normality hypothesis was rejected, and non-parametric methods were used. A Friedman test was carried out in order to evaluate significant differences between times of accelerometric recording in both experiments (sham and CRET). When significant differences were detected, a post-hoc test was performed (Wilcoxon rank test). The comparisons between sham and CRET experiments in each of the times were made with a Mann-Whitney test.

The differences in power percentages in the three corporal axes were evaluated with the Q of Cochran. Correlations between the different variables at the walk and at the trot were calculated with a Spearman Rank R Correlation, considering the data obtained at all the measurements’ times.

All these tests were performed separately for the two gaits and for the two locations of the accelerometric device (PECT and SML). Comparisons between gaits and locations of the accelerometric device were out of the scope of the research and were not carried out. Significant differences in ambient temperatures and relative humidity were evaluated with a repeated measures ANOVA. Values of *P* < 0.05 were considered significant.

## Results

None of the horses showed any negative side effects during the study. On the contrary, a relaxion state was noted during the application of CRET, with head lowering, fall of the lower lip… Because all the procedures were carried out in two acclimatized rooms (exam and treadmill rooms), significant differences in the environmental conditions when comparing the times of the accelerometric recordings and the days of the study were not found. Mean ambient temperature of 25.3 °C (range 22.1 and 29.4 °C) and relative humidity of 45% (range 32 and 51%) were recorded.

Significant differences were not found between the different times during the sham experiment, for any of the accelerometric parameters.

### Changes in energetic parameters

**Fig. 1:**
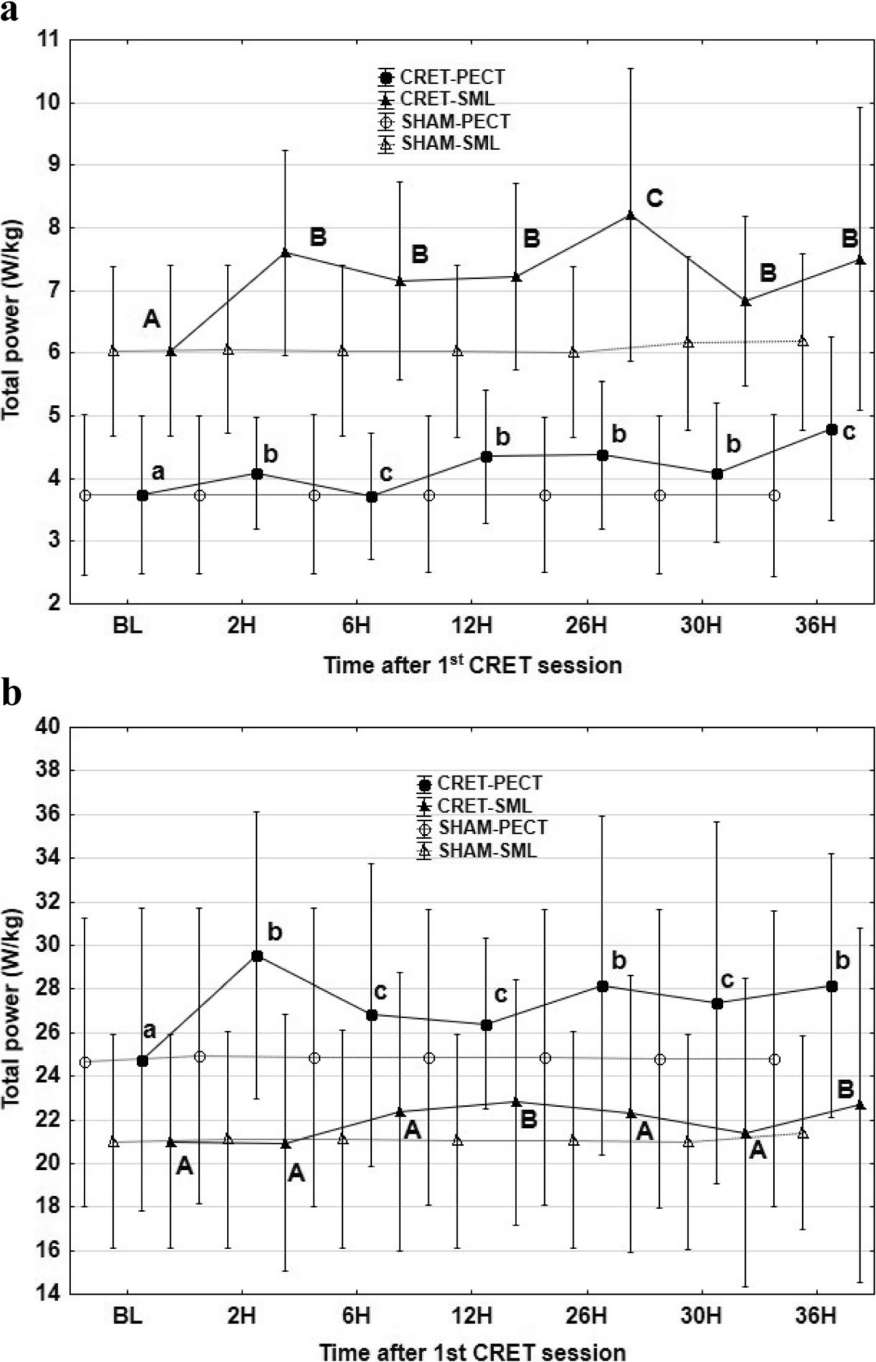
Mean values and standard deviations of total power at walk (**a**) and trot (**b**), with the accelerometer in the sternum, PECT (●) and in sacrum midline, SML (▲), at defined times after the first session of capacitive resistive electric transfer session compared to a sham procedure (Values with different letters are significantly different from each other between times of accelerometric recordings) *P* < 0.05. (lowercase letters indicate significant differences with the accelerometer in the sternum; capital letters indicate significant differences with the accelerometer in the sacrum midline)

Total power increased from BL at walk and at trot, with the accelerometer located in both anatomical regions (Fig. 1). At walk, the highest TP values were observed at 36 h (accelerometer in PECT position) and at 26 h (accelerometer in SML position). At trot, the highest values were found at 2 h (PECT) and at 12 and 36 h (SML), as shown in Fig. 1.

**Fig. 2:**
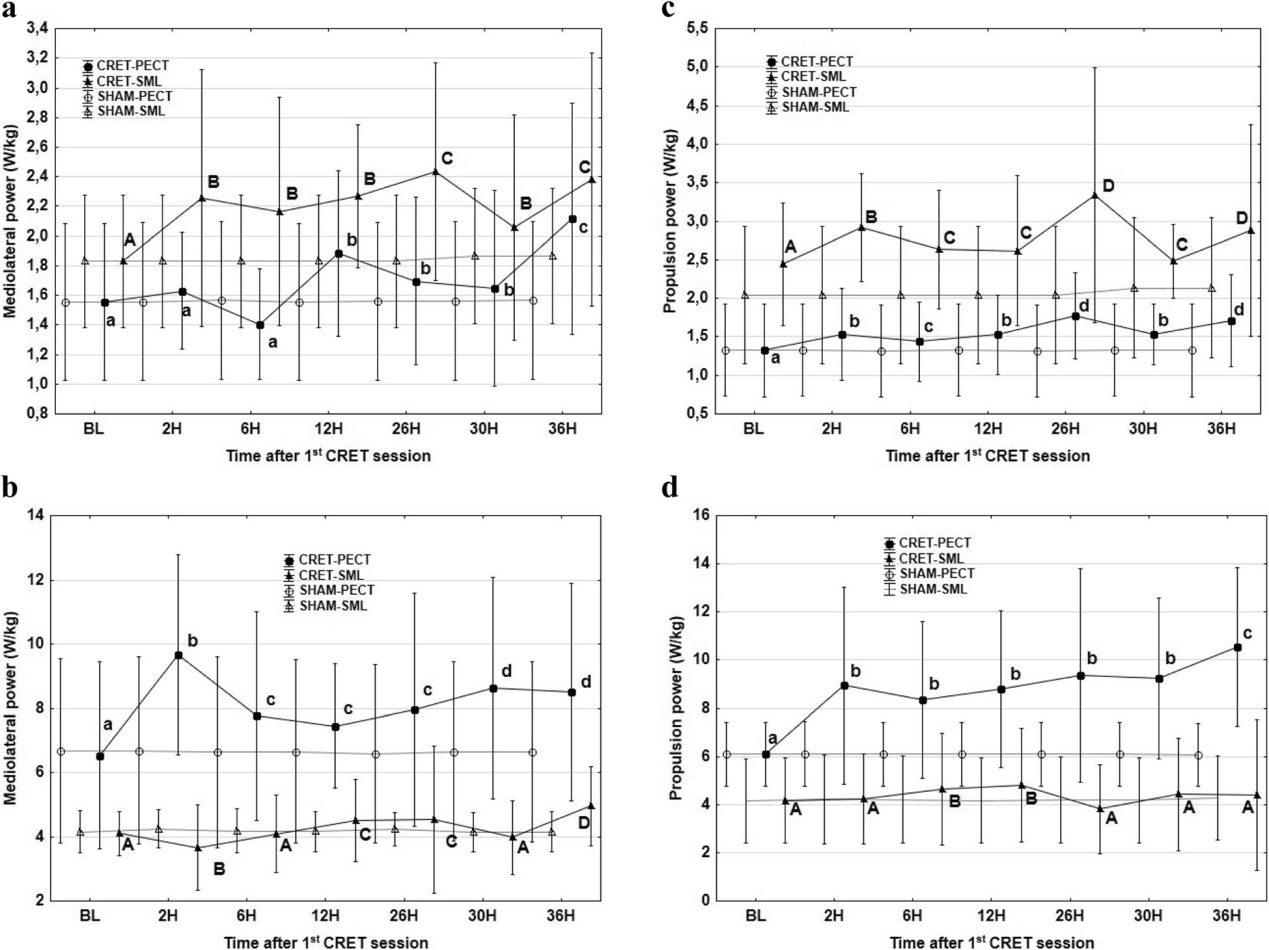
Mean values and standard deviations of mediolateral power at walk (**a**) and trot (**b**) and propulsion power at walk (**c**) and trot (**d**) with the accelerometer in the sternum, PECT (●) and in sacrum midline, SML (▲), at defined times after the first session of capacitive resistive electric transfer session compared to a sham procedure (Values with different letters are significantly different from each other between times of accelerometric recordings) *P* < 0.05. (lowercase letters indicate significant differences with the accelerometer in the sternum; capital letters indicate significant differences with the accelerometer in the sacrum midline)

Figure 2 presents data of MLP and PP. In most of the recording times, MLP and PP values increased significantly from the BL values. An accumulative effect appeared to happen in MLP and PP at walk, with the accelerometer in both anatomical positions. Such accumulative effect did not appear to happen in MLP at trot with the accelerometer in PECT position neither in PP at trot, with the accelerometer in SML (Fig. 2).

**Fig. 3:**
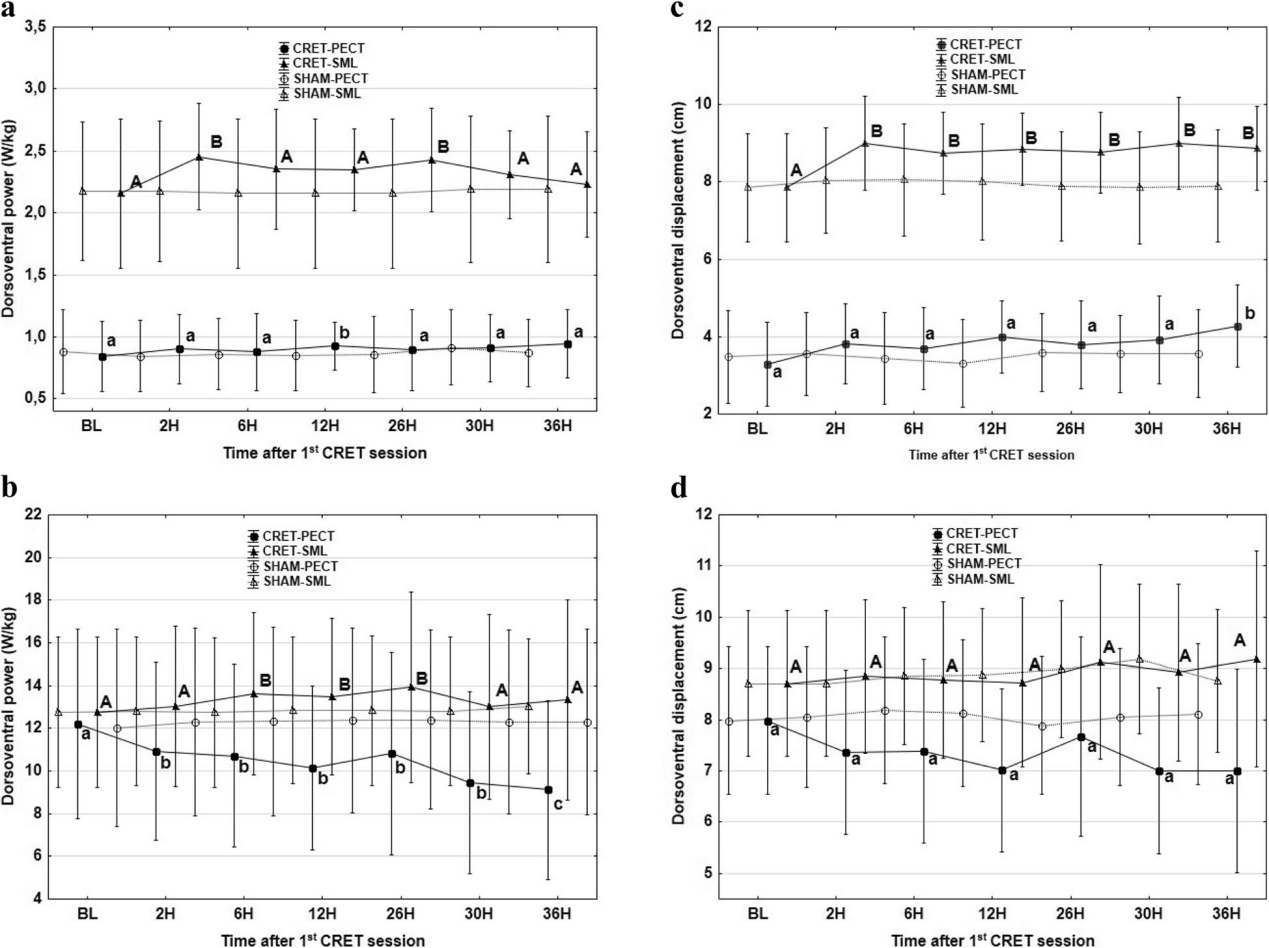
Mean values and standard deviations of dorsoventral power at walk (**a**) and trot (**b**) and dorsoventral displacement at walk (**c**) and trot (**d**) with the accelerometer in the sternum, PECT (●) and in sacrum midline, SML (▲), at defined times after the first session of capacitive resistive electric transfer session compared to a sham procedure (Values with different letters are significantly different from each other between times of accelerometric recordings) *P* < 0.05. (lowercase letters indicate significant differences with the accelerometer in the sternum; capital letters indicate significant differences with the accelerometer in the sacrum midline)

Dorsoventral power increased at walk, reaching the maximum values at 12 h with the accelerometer in PECT position and at 2 h and 26 h with the accelerometer in SML (Fig. 3). Similarly, there was an increase in DVD at walk, at 36 h with the accelerometer in PECT and from 2 to 36 h with the accelerometer in SML. On the contrary, there was a decrease in DVP, together a non-significant trend (*p* < 0.067) towards a reduction in DVD at trot with the accelerometer in PECT (Fig. 3).

Percentages of the distribution of TP in the three body axes (PML, PP and DVP) for the accelerometer fixed in PECT and SML are presented for the walk and trot in Tables 1 and 2 respectively. At walk, DVP% decreased at 26 h with the accelerometer in PECT and at 36 h with the accelerometer in SML. MLP% decreased at 6 h but increased at 36 h with the accelerometer in PECT, whereas PP% increased at 26 h with the accelerometer in PECT and at 2, 26 and 36 h with the accelerometer in SML (Table 1). At trot, DVP% decreased at 2, 30 and 36 h with the accelerometer in PECT. MLP% increased at 2 h with the accelerometer in PECT and PP% increased at 36 h with the accelerometer in PECT but decreased at 36 h with the accelerometer in SML (Table 2).

**Table 1:** Mean and standard deviation of the percentages of dorsoventral (DVP), propulsion (PP) and mediolateral (MLP) powers at walk, with the accelerometer in the pectoral region and in the sacrum midline at defined times after a sham procedure and after the first session of capacitive resistive electric transfer session (Values with different letters are significantly different from each other between times of accelerometric recordings) *P* < 0.05

Time	DVP%	MLP%	PP%	DVP%	%MLP	PP%
Accelerometer located in the pectoral region (PECT)						
	SHAM procedure			After CRET application		
BL	24.82 ± 7.10 ^a^	41.69 ± 4.91 ^a^	33.49 ± 5.25 ^a^	23.43 ± 6.12 ^a^	41.61 ± 4.91 ^a^	34.96 ± 5.40 ^a^
2 h	23.50 ± 6.17 ^a^	41.75 ± 5.01 ^a^	34.75 ± 5.49 ^a^	22.85 ± 7.28 ^a^	40.01 ± 4.85 ^a^	37.14 ± 8.96 ^a^
6 h	24.03 ± 6.77 ^a^	41.78 ± 4.43 ^a^	34.19 ± 6.39 ^a^	24.57 ± 5.69 ^a^	37.65 ± 4.51 ^b^	37.78 ± 7.33 ^a^
12 h	23.62 ± 6.53 ^a^	42.39 ± 4.83 ^a^	33.99 ± 5.50 ^a^	22.34 ± 6.12 ^a^	42.93 ± 4.58 ^a^	34.73 ± 6.07 ^a^
26 h	23.78 ± 8.52 ^a^	41.62 ± 4.99 ^a^	34.60 ± 6.39 ^a^	20.98 ± 6.22 ^b^	38.45 ± 4.46 ^a^	40.57 ± 6.64 ^b^
30 h	25.72 ± 7.36 ^a^	42.82 ± 4.72 ^a^	31.46 ± 8.58 ^a^	22.85 ± 6.11 ^a^	39.48 ± 6.02 ^a^	37.67 ± 5.27 ^a^
36 h	24.54 ± 6.56 ^a^	43.09 ± 5.54 ^a^	32.37 ± 5.95 ^a^	21.05 ± 7.41 ^a^	43.37 ± 4.25 ^b^	35.48 ± 3.98 ^a^
Accelerometer located in the sacrum midline (SML)						
	SHAM procedure			After CRET application		
BL	36.31 ± 7.12 ^a^	31.06 ± 9.59 ^a^	32.63 ± 8.17 ^a^	35.80 ± 7.96 ^a^	31.44 ± 9.68 ^a^	32.76 ± 8.09 ^a^
2 h	35.99 ± 7.13 ^a^	31.27 ± 9.63 ^a^	32.74 ± 8.29 ^a^	32.62 ± 3.19 ^a^	29.87 ± 6.15 ^a^	37.51 ± 6.22 ^b^
6 h	36.01 ± 8.13 ^a^	31.44 ± 9.65 ^a^	32.55 ± 8.02 ^a^	33.21 ± 4.49 ^a^	30.11 ± 6.92 ^a^	36.68 ± 6.14 ^a^
12 h	35.89 ± 8.12 ^a^	31.48 ± 9.68 ^a^	32.63 ± 8.08 ^a^	33.02 ± 3.96 ^a^	31.55 ± 4.03 ^a^	35.43 ± 6.53 ^a^
26 h	35.92 ± 7.91 ^a^	31.40 ± 9.82 ^a^	32.68 ± 8.13 ^a^	30.92 ± 6.32 ^b^	30.89 ± 6.93 ^a^	38.19 ± 8.96 ^b^
30 h	35.50 ± 7.06 ^a^	31.06 ± 10.3 ^a^	33.44 ± 9.30 ^a^	34.23 ± 4.30 ^a^	29.32 ± 6.17 ^a^	36.45 ± 4.52 ^a^
36 h	35.22 ± 7.12 ^a^	31.45 ± 10.2 ^a^	33.33 ± 8.29 ^a^	30.85 ± 5.05 ^b^	31.72 ± 6.21 ^a^	37.43 ± 5.58 ^b^

**Table 2:** Mean and standard deviation of the percentages of dorsoventral (DVP), propulsion (PP) and mediolateral (MLP) powers at trot, with the accelerometer in the pectoral region and in the sacrum midline at defined times after a sham procedure and after the first session of capacitive resistive electric transfer session (Values with different letters are significantly different from each other between times of accelerometric recordings) *P* < 0.05

Time	DVP%	MLP%	PP%	DVP%	MLP%	PP%
Accelerometer located in the pectoral region (PECT)						
	SHAM procedure			After CRET application		
BL	47.80 ± 10.7 ^a^	26.50 ± 8.87 ^a^	25.70 ± 6.38 ^a^	48.44 ± 9.04 ^a^	25.51 ± 5.23 ^a^	26.05 ± 4.130 ^a^
2 h	48.47 ± 9.16 ^a^	26.25 ± 8.70 ^a^	25.28 ± 5.40 ^a^	37.74 ± 14.70 ^b^	32.14 ± 4.35 ^b^	30.12 ± 12.85 ^a^
6 h	48.76 ± 9.50 ^a^	26.32 ± 8.89 ^a^	24.94 ± 5.47 ^a^	40.64 ± 15.85 ^a^	28.11 ± 6.70 ^a^	31.25 ± 11.11 ^a^
12 h	49.00 ± 9.21 ^a^	26.28 ± 8.80 ^a^	24.72 ± 5.20 ^a^	38.41 ± 13.74 ^a^	28.11 ± 6.70 ^a^	33.48 ± 18.06 ^a^
26 h	49.30 ± 8.18 ^a^	26.28 ± 8.78 ^a^	25.42 ± 5.14 ^a^	40.40 ± 17.90 ^a^	26.90 ± 7.54 ^a^	32.70 ± 12.28 ^a^
30 h	48.85 ± 8.64 ^a^	26.38 ± 8.89 ^a^	24.77 ± 5.37 ^a^	34.67 ± 12.88 ^b^	30.72 ± 4.81 ^a^	34.61 ± 11.70 ^a^
36 h	48.82 ± 8.71 ^a^	26.36 ± 8.90 ^a^	24.82 ± 5.24 ^a^	32.19 ± 12.88 ^b^	29.61 ± 5.52 ^a^	38.20 ± 13.08 ^b^
Accelerometer located in the sacrum midline (SML)						
	SHAM procedure			After CRET application		
BL	59.85 ± 7.09 ^a^	20.93 ± 5.71 ^a^	19.22 ± 5.39 ^a^	59.83 ± 7.17 ^a^	20.65 ± 5.85 ^a^	19.52 ± 5.44 ^a^
2 h	59.99 ± 7.13 ^a^	22.19 ± 11.8 ^a^	17.82 ± 5.56 ^a^	62.45 ± 7.29 ^a^	18.24 ± 7.07 ^a^	19.31 ± 3.63 ^a^
6 h	59.65 ± 7.14 ^a^	20.94 ± 5.62 ^a^	19.41 ± 5.44 ^a^	61.20 ± 6.06 ^a^	19.04 ± 4.57 ^a^	19.76 ± 4.81 ^a^
12 h	60.37 ± 7.18 ^a^	21.47 ± 9.08 ^a^	18.16 ± 5.44 ^a^	59.10 ± 6.61 ^a^	20.96 ± 7.15 ^a^	19.94 ± 6.51 ^a^
26 h	60.15 ± 7.05 ^a^	21.93 ± 10.4 ^a^	17.92 ± 5.40 ^a^	62.27 ± 7.40 ^a^	21.13 ± 8.66 ^a^	16.60 ± 4.62 ^a^
30 h	60.21 ± 7.11 ^a^	21.79 ± 11.9 ^a^	18.00 ± 5.45 ^a^	60.78 ± 3.98 ^a^	19.56 ± 5.54 ^a^	19.86 ± 4.72 ^a^
36 h	60.64 ± 4.92 ^a^	20.28 ± 5.41 ^a^	19.08 ± 4.71 ^a^	58.96 ± 5.70 ^a^	23.83 ± 7.29 ^a^	17.21 ± 6.36 ^b^

### Changes in coordination parameters

Coordination parameters are presented in Tables 3 and 4 for walk and trot respectively. REG at walk increased from BL at 26, 30 and 36 h with the accelerometer in PECT and at 30 and 36 h with the accelerometer in SML. The only significant change in this parameter at trot was observed at 30 h with the accelerometer in PECT, showing values higher than BL. SYM showed a reduction from BL at walk at 6, 12 and 36 h with the accelerometer at SML and at 6 and 26 h at trot with the accelerometer in PECT. Significant changes in STA were not found (Tables 3 and 4).

**Table 3:** Mean and standard deviation of the stride regularity (REG), symmetry (SYM) and stability (STA) at walk, with the accelerometer in the pectoral region and in the sacrum midline at defined times after a sham procedure and after the first session of capacitive resistive electric transfer session (Values with different letters are significantly different from each other between times of accelerometric recordings) *P* < 0.05

Time	REG	SYM	STA	REG	SYM	STA
Accelerometer in the pectoral region (PECT)						
	SHAM procedure			After CRET application		
BL	244.3 ± 51.37 ^a^	170.1 ± 57.21 ^a^	414.4 ± 73.97 ^a^	244.2 ± 51.03 ^a^	176.3 ± 54.29 ^a^	420.8 ± 89.96 ^a^
2 h	252.1 ± 54.22 ^a^	169.6 ± 47.21 ^a^	421.7 ± 81.03 ^a^	257.8 ± 63.33 ^a^	176.4 ± 52.49 ^a^	434.2 ± 97.85 ^a^
6 h	246.4 ± 48.88 ^a^	175.9 ± 57.91 ^a^	422.3 ± 77.16 ^a^	246.6 ± 59.25 ^a^	187.9 ± 57.49 ^a^	434.5 ± 82.50 ^a^
12 h	249.3 ± 53.56 ^a^	170.0 ± 48.88 ^a^	419.3 ± 74.86 ^a^	256.0 ± 63.74 ^a^	183.8 ± 62.56 ^a^	439.7 ± 93.91 ^a^
26 h	253.1 ± 54.42 ^a^	176.4 ± 55.44 ^a^	419.3 ± 74.86 ^a^	264.3 ± 55.54 ^b^	177.1 ± 64.03 ^a^	441.4 ± 91.78 ^a^
30 h	243.9 ± 54.79 ^a^	176.6 ± 51.45 ^a^	429.5 ± 71.56 ^a^	265.8 ± 53.75 ^b^	168.7 ± 45.32 ^a^	434.4 ± 79.42 ^a^
36 h	245.4 ± 50.45 ^a^	173.3 ± 54.23 ^a^	418.7 ± 77.51 ^a^	262.1 ± 58.48 ^b^	186.6 ± 49.70 ^a^	448.7 ± 94.73 ^a^
Accelerometer in the sacrum midline (SML)						
	SHAM procedure			After CRET application		
BL	348.8 ± 66.08 ^a^	236.4 ± 45.97 ^a^	585.2 ± 75.05 ^a^	305.4 ± 51.76 ^a^	243.9 ± 59.63 ^a^	555.1 ± 99.99 ^a^
2 h	350.6 ± 60.82 ^a^	217.6 ± 46.63 ^a^	568.2 ± 81.12 ^a^	337.0 ± 58.75 ^a^	225.4 ± 53.47 ^a^	562.4 ± 81.47 ^a^
6 h	343.7 ± 55.30 ^a^	217.4 ± 42.92 ^a^	561.1 ± 73.47 ^a^	331.1 ± 66.72 ^a^	202.3 ± 41.74 ^b^	533.4 ± 72.15 ^a^
12 h	340.6 ± 60.10 ^a^	232.1 ± 49.36 ^a^	572.7 ± 76.82 ^a^	337.0 ± 51.18 ^a^	195.5 ± 42.32 ^b^	532.4 ± 62.83 ^a^
26 h	343.9 ± 61.55 ^a^	223.3 ± 41.90 ^a^	567.2 ± 82.80 ^a^	331.1 ± 75.43 ^a^	218.8 ± 50.82 ^a^	549.8 ± 93.12 ^a^
30 h	342.5 ± 58.73 ^a^	206.3 ± 44.94 ^a^	548.8 ± 73.34 ^a^	345.7 ± 52.16 ^b^	223.1 ± 47.69 ^a^	568.8 ± 82.19 ^a^
36 h	348.4 ± 54.77 ^a^	220.4 ± 50.36 ^a^	568.8 ± 80.17 ^a^	356.1 ± 40.80 ^b^	192.1 ± 41.52 ^b^	548.2 ± 51.42 ^a^

**Table 4:** Mean and standard deviation of the stride regularity (REG), symmetry (SYM) and stability (STA) at trot, with the accelerometer in the pectoral region and in the sacrum midline at defined times after a sham procedure and after the first session of capacitive resistive electric transfer session (Values with different letters are significantly different from each other between times of accelerometric recordings) *P* < 0.05

Time	REG	SYM	STA	REG	SYM	STA
Accelerometer in the pectoral region (PECT)						
	SHAM procedure			After CRET application		
BL	374.2 ± 50.68 ^a^	240.6 ± 54.07 ^a^	614.8 ± 80.87 ^a^	377.4 ± 59.31 ^a^	252.2 ± 48.15 ^a^	620.6 ± 89.98 ^a^
2 h	374.5 ± 45.52 ^a^	243.7 ± 56.20 ^a^	618.2 ± 62.25 ^a^	369.5 ± 50.75 ^a^	252.4 ± 61.14 ^a^	621.9 ± 74.39 ^a^
6 h	376.3 ± 41.18 ^a^	253.7 ± 46.41 ^a^	630.0 ± 80.97 ^a^	375.6 ± 38.66 ^a^	227.5 ± 30.76 ^b^	603.1 ± 53.45 ^a^
12 h	378.5 ± 42.04 ^a^	242.8 ± 50.25 ^a^	621.2 ± 71.72 ^a^	381.2 ± 46.76 ^a^	240.9 ± 50.02 ^a^	622.0 ± 73.09 ^a^
26 h	373.6 ± 40.42 ^a^	243.8 ± 50.65 ^a^	617.3 ± 56.30 ^a^	369.6 ± 51.13 ^a^	229.4 ± 52.99 ^b^	598.9 ± 83.47 ^a^
30 h	376.1 ± 41.78 ^a^	249.5 ± 42.82 ^a^	625.6 ± 56.60 ^a^	368.0 ± 54.11 ^a^	239.8 ± 63.50 ^a^	607.8 ± 99.57 ^a^
36 h	371.1 ± 43.33 ^a^	253.7 ± 55.77 ^a^	624.8 ± 68.10 ^a^	370.8 ± 43.57 ^a^	239.1 ± 48.74 ^a^	609.9 ± 71.01 ^a^
Accelerometer in the sacrum midline (SML)						
	SHAM procedure			After CRET application		
BL	366.9 ± 42.80 ^a^	208.2 ± 50.97 ^a^	575.1 ± 67.65 ^a^	337.7 ± 55.80 ^a^	236.1 ± 61.27 ^a^	573.8 ± 88.38 ^a^
2 h	360.7 ± 41.54 ^a^	214.6 ± 51.10 ^a^	575.3 ± 61.50 ^a^	365.7 ± 59.27 ^a^	208.8 ± 38.46 ^a^	574.5 ± 80.22 ^a^
6 h	363.8 ± 38.81 ^a^	214.9 ± 48.18 ^a^	578.7 ± 61.12 ^a^	349.5 ± 48.49 ^a^	211.0 ± 42.31 ^a^	560.5 ± 62.54 ^a^
12 h	360.1 ± 39.26 ^a^	218.6 ± 43.07 ^a^	578.7 ± 59.58 ^a^	371.6 ± 36.63 ^a^	208.1 ± 54.77 ^a^	579.8 ± 71.38 ^a^
26 h	363.4 ± 38.54 ^a^	227.9 ± 51.39 ^a^	591.3 ± 62.60 ^a^	342.6 ± 59.44 ^a^	215.1 ± 73.22 ^a^	557.6 ± 82.15 ^a^
30 h	368.4 ± 33.36 ^a^	227.1 ± 42.40 ^a^	595.5 ± 64.26 ^a^	375.4 ± 42.57 ^b^	224.1 ± 44.46 ^a^	599.5 ± 62.52 ^a^
36 h	363.2 ± 32.24 ^a^	225.3 ± 50.64 ^a^	588.5 ± 61.46 ^a^	368.8 ± 32.78 ^a^	213.9 ± 41.41 ^a^	582.7 ± 54.46 ^a^

### Changes in stride spatiotemporal parameters

**Fig. 4:**
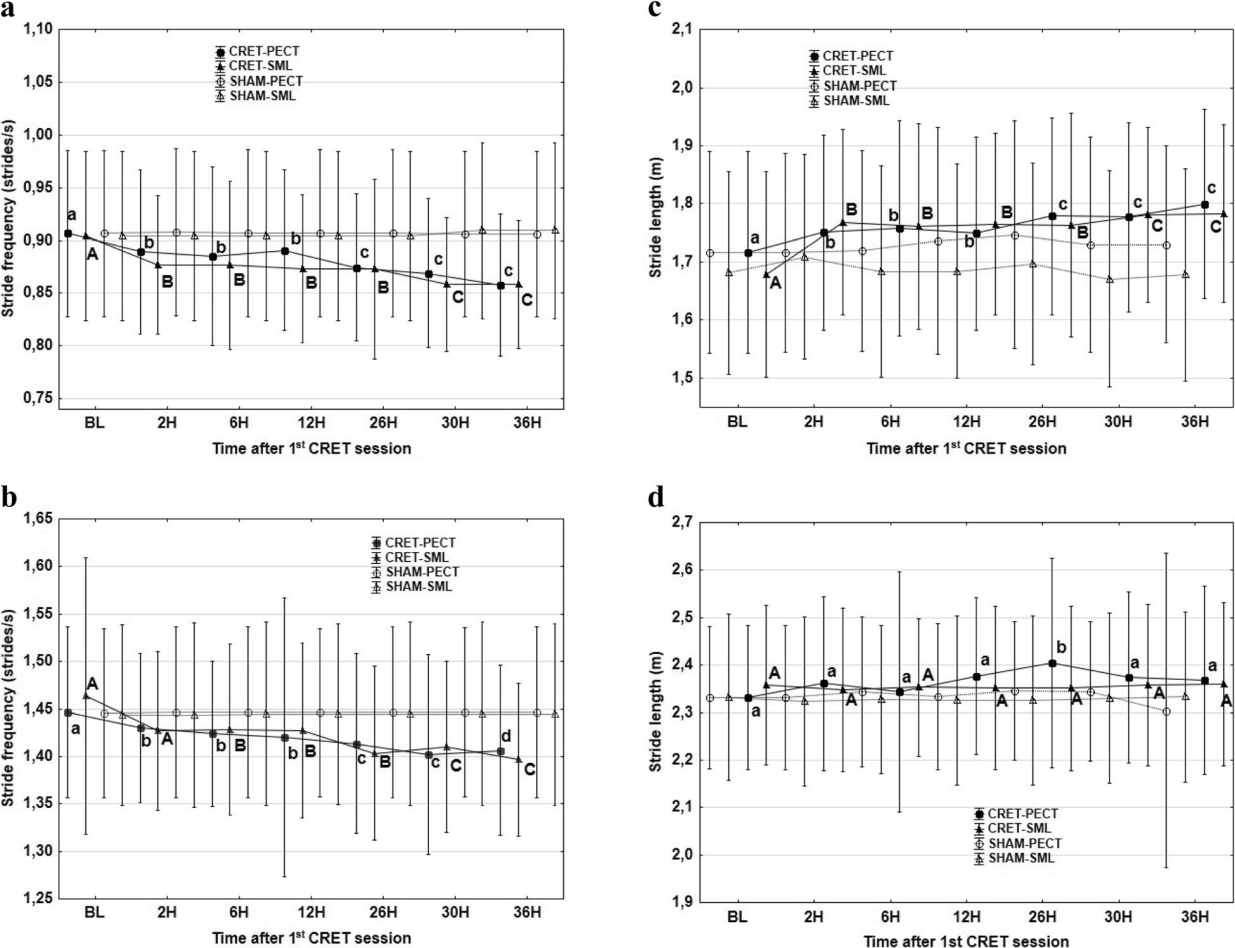
Mean values and standard deviations of stride frequency at walk (**a**) and trot (**b**) and stride length at walk (**c**) and trot (**d**) with the accelerometer in the sternum, PECT (●) and in sacrum midline, SML (▲), at defined times after the first session of capacitive resistive electric transfer session compared to a sham procedure (Values with different letters are significantly different from each other between times of accelerometric recordings) *P* < 0.05. (lowercase letters indicate significant differences with the accelerometer in the sternum; capital letters indicate significant differences with the accelerometer in the sacrum midline)

Changes in SF and SL are shown in Fig. 4. Significant decreases in SF and increases in SL were found for the two gaits and with the accelerometer in PECT and SML. SF and SL decreased and increased respectively during the second day (times 26, 30 and 36 h) compared with the first day (2, 6 and 12 h). The exception of these results was the SL at trot with the accelerometer at SML, that did not experience any significant change (Fig. 4).

### Correlations

Correlations between the different parameters are presented in Table 5.

**Table 5:** Spearman rank order correlations between energetic stride parameters (total power and power distribution in the three body axis) and, coordination parameters and stride spatiotemporal parameters in 9 horses after two sessions of capacitive resistive electric transfer applied on two consecutive days. ^a^ Correlation coefficient significant at *P* < 0.05. (TP: total power; DVP: dorsoventral power; PP: propulsion power; MLP: mediolateral power; DDV: dorsoventral power)

	TP	DVP	PP	MLP	DDV
Symmetry	0.296	0.315	0.216	0.730	0.284
Regularity	0.541	0.592	0.422	0.430	0.537
Stability	0.340	0.505	0.315	0.590	0.433
Stride length	0.887 ^a^	0.707	0.917 ^a^	0.690	0.491
Stride frequency	0.825 ^a^	0.735	0.765	0.732	0.136

## Discussion

The current research assessed the effect of CRET, a RF at 448 kHz, on the locomotor pattern of horse evaluated on a treadmill. We confirmed two of the three proposed hypotheses in our study. The first hypothesis was that a session of CRET would modify TP and would redistribute this TP in the three body axes. We found that the application of CRET resulted in an increased TP and consequently, the absolute values of the powers in the three body axes rose, except for DVP% at trot with the accelerometer in PECT. Furthermore, when these values were examined as percentages of TP, a reduction in DVP%, together with an increase in PP% and in MLP%, were detected. Our second hypothesis was that, changes in TP would affect the stride spatiotemporal variables. In this way, we found that CRET led to an increase in SL together with a reduction in SF. The third hypothesis, however, could not completely been confirmed. We hypothesized that an accumulative effect of the CRET would be observed when two sessions of CRET were applied on two consecutive days. Although an accumulative effect was found in some of the studied variables (SF, SL), such effect was not clear in others (TP, DVP, MLP, PP).

### Changes in energetic parameters

Previous studies performed in human beings have shown that CRET exerts diverse thermophysiological responses which have been considered the basis of its therapeutic effects [12, 13]. The main underlying mechanism is considered to be the rise in temperature associated with electromagnetic energy, which triggers oscillation and friction forces of charged molecules, such as proteins and ions. This increase in temperature has been described at a superficial level (10–15 mm, which is believed to reflect the state of the subcutaneous adipose tissue and fascia), but also at a deeper level (20–30 mm, which is supposed to reflect the state of the muscle layer) [7, 8, 13]. The mild elevation of the temperature achieved in these studies was enough to accelerate metabolic activity and to improve local circulation. It is plausible to assume that these same effects would have occurred in the horses in the present investigation, leading to an improvement in muscle function and hence the significant increase in TP. However, the above mentioned authors conducted sessions of 15 min of duration (5 min capacitive; 10 min resistive), whereas in our study a shorter time of application was used (2 min capacitive; 4–5 min resistive), even CRET was applied in greater anatomical areas in the horses, and therefore, affecting more muscles.

The beneficial effects of a slight elevation in muscle temperature before exercise on muscle functionality during exercise have been reported in human athletes [14, 15], being one of the reasons for warming and stretching before exercise. Unfortunately, in the current research we have documented the direct consequence of the application of CRET technique on muscle power (increased TP) and on locomotor pattern, but reliable hypotheses about its mechanisms of action cannot be provided.

The increase in TP caused by CRET led to an increase in power in the three body axes in the horses. Despite this affirmation, different findings were observed when evaluating the changes in the percentage of the three powers. CRET resulted in a deviation of TP towards the longitudinal and mediolateral axes at the expense of the dorsoventral axis. In our opinion, these findings might be partially due to the fact that the horses were exercised on a treadmill. In an early study, Buchner et al. [9] described that the vertical displacement of the stride was smaller in horses exercised on a treadmill compared to asphalt. More recently, we have reported that DVD and DVP were lower in horses exercised on a treadmill compared to a soft surface (training arena) and on a hard surface (asphalt) [16]. The horses of the current report presented a reduction in DVP%, but DDV increased, albeit only at the walk. The combined consideration of these results seems to reflect that the decrease in DVP% was due to a redistribution of the TP rather than to a real reduction of DVP. An investigation of the CRET effects on these accelerometric parameters evaluated on a training surface will be concluded soon and might provide an explanation on whether this power redistribution was due to the effect of CRET or on the contrary, it was a consequence of the locomotor pattern on the treadmill.

### Changes in coordination parameters

REG is an accelerometric specific variable referring to stride to stride variability, SYM refers to the similarity between left and the right phases of the stride and STA is described as an indicator of gait quality [4]. The increase in REG observed mainly at walk was compatible with a more homogenous locomotor pattern. In fact, lameness is characterized by a reduction in REG [17]. The reason for the reduction experimented by SYM is unknown.

### Changes in stride spatiotemporal parameters

One of the most consistent response to CRET in the current research was the increase in SL and consequently, the reduction in SF, as the treadmill velocity remained invariable. These results agreed with those reported by Duñabeitia et al. [6], who described an increase in step length and a reduction in step frequency in recreational runners after CRET. These changes were attributed to the thermophysiological responses caused by CRET. In our study, and according to the correlation analysis, the main determinants of the longer SL were the increases in TP and particularly in PP.

In addition, a summing effect appeared to happen in the second session of CRET in these parameters, since SL was longer on the second day (26, 30 and 36 h after the first session) compared to the first day (2, 6 and 12 h). The duration of the increased temperature and other physiological effects caused by CRET have not been defined exactly, and skin, superficial and deep temperature, blood flow and hemoglobin saturation, have been measured up to only to 30 min post-application [7, 8, 13]. Moreover, several in vitro studies have shown that CRET application also exerts non-thermal effects, consisting of a modulation of cellular activity and changes in membrane transport [18, 19]. In our study, the changes in the locomotor pattern were maintained for at least 12 h and in some cases (SL and SF), they even changed more intensely after the second session of CRET.

A limitation of our study was that we were unable to measure skin and muscle temperature in response to CRET, that would have provided relevant information regarding the mechanism of action of this technique. Superficial temperature can be measured with infrared thermography, but we could not measure it because the skin was moistened, and a conductive gel was applied between the electrode and the skin surface. Muscle temperature measurements would have required implantable thermistor probes inserted into the muscles. However, this technique was not used because electromagnetic field could have directly heated the measurement device, leading to erroneous temperature recordings. In addition, some of the horses included in the research belonged to owners that would not have approved the use of invasive methods. Furthermore, the invasive procedure would trigger an inflammatory response which potentially might affect the local physiological activity.

We chose to carry out the study on a treadmill because of the higher standardization of the exercise in terms of surface and speed compared to a track. However, it is well established that equine locomotion significantly differs between treadmill and track [9, 20]. Possibly treadmill exercise affected DVP, PP and DVD, and in addition, the animals could not freely choose the velocity during exercise. It is interesting to speculate that, whether the animals had freely chosen the exercise velocity, the locomotor changes associated with CRET could have been even more intense. This aspect deserves future research.

## Conclusions

Our study demonstrated that application of CRET on the horse significantly modifies the accelerometric pattern of the horse evaluated on a treadmill, leading to increased muscle power, longer SL and lower SF. Further, the observed modifications (increases of TP, DVP, PP, MLP, DVD, SL), according to previous studies performed in different equine athletes, such as racing thoroughbreds [2], jumpers [1] and dressage [3], appear to relate to better sport performances. However, the evaluation of the effects of these locomotor changes on performance was beyond the scope of this article, but they should be studied in a next future for horses competing in different sport disciplines.

## Data Availability

Data and materials are available from the corresponding author upon reasonable request.

## Abbreviations

BL: Baseline
CAP: Capacitive
CRET: Capacitive resistive electric transfer
DVD: Dorsoventral displacement
FFT: Fast Fourier transformation
MLP: Mediolateral power
PECT: Pectoral location
PP: Propulsive power
REG: Stride regularity
RES: Resistive
SF: Stride frequency
SL: Stride length
SML: Midline of the sacrum region
STAB: Stride stability
SYM: Stride symmetry
TE: Treadmill exercise
TP: Total power
V: Velocity
